# Supplementary material for: Recurrent intragenic rearrangements of EGFR and BRAF in soft tissue tumors of infants
Source: Nat Commun. 2018 Jun 18;9:2378. doi: 10.1038/s41467-018-04650-6 (PMC6006309; doi:10.1038/s41467-018-04650-6)
Supplement: Supplementary file 1 — Supplementary Information [file 41467_2018_4650_MOESM1_ESM.pdf]

Supplementary Information

Recurrent intragenic rearrangements of *EGFR* and *BRAF* in soft tissue tumors of infants

Wegert *et al*.

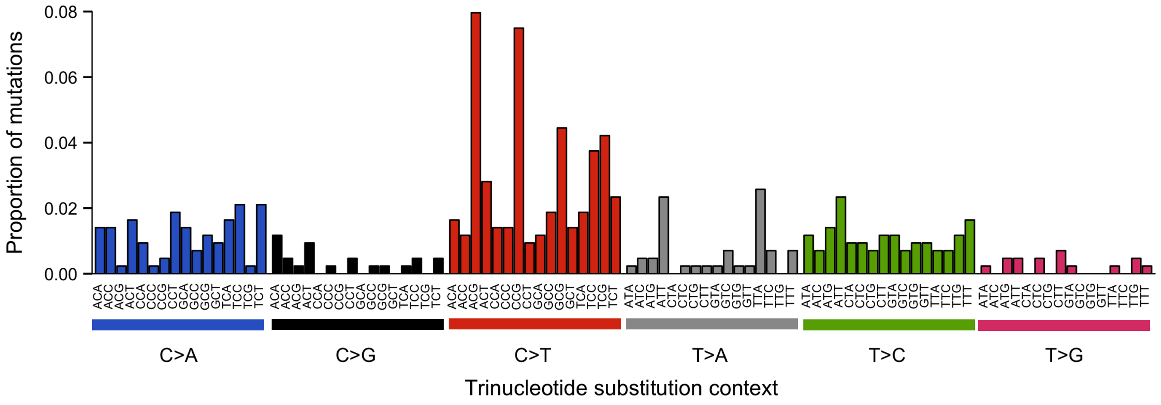

Supplementary Figure 1 | Trinucleotide context of substitution mutations in 10 classical congenital mesoblastic nephroma genomes. The x-axis indicates the 96 classes of substitution according to trinucleotide context; proportion of substitution mutations in each class is shown on the y-axis.

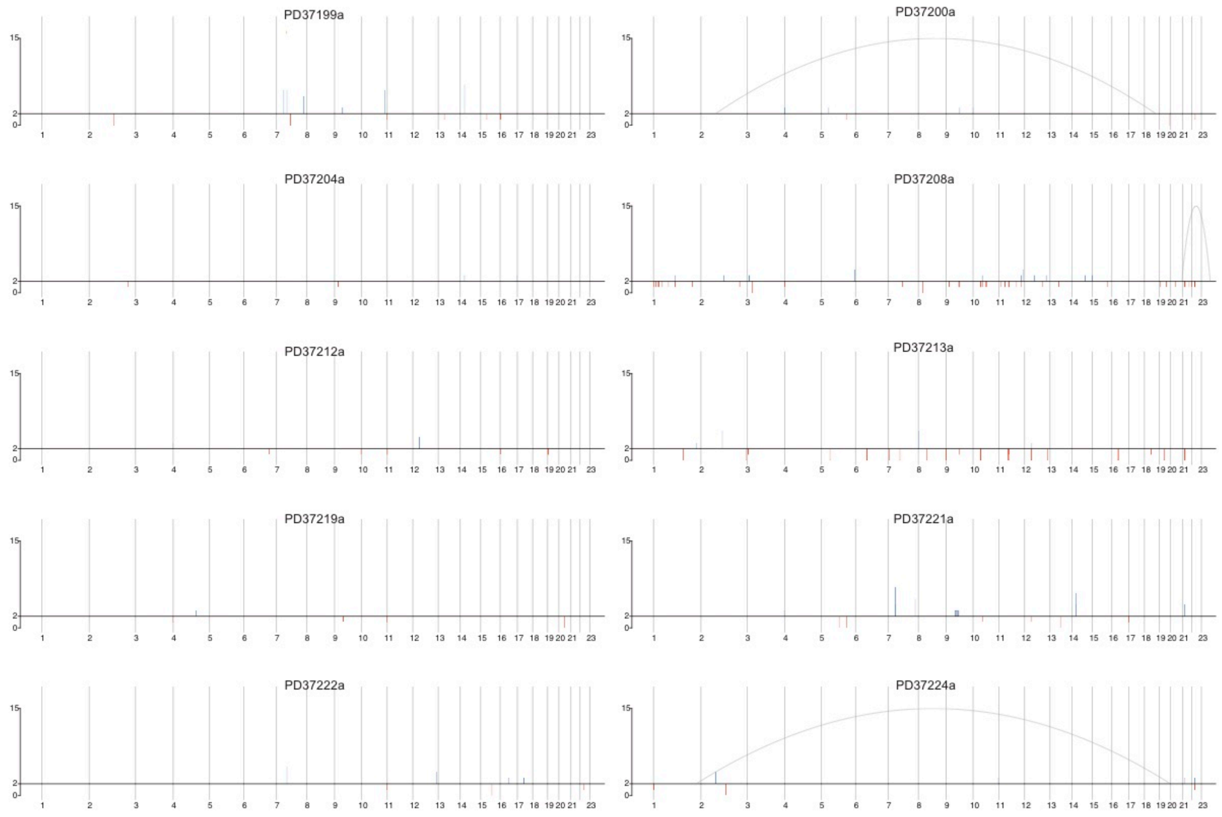

Supplementary Figure 2 | Structural changes in 10 congenital mesoblastic nephroma genomes. Copy number profile and rearrangements are shown for 10 classical histology CMN lacking the canonical *ETV6-NTRK3* fusion. Absolute copy number is represented on the y-axis; chromosomes are indicated on the x-axis. Blue and red horizontal segments represent copy number gains and losses, respectively. Grey lines indicate start and end of translocations.

Supplementary Table 1 | Primer sequences

Target	Primer name	Sequence
***EGFR*-ITD**	hEGFR-ITD1 [1, 3]	CGACGAGTACCTCATCCCAC
	hEGFR-D2 [1]	AGCTTGGTTGGGAGCTTCTC
	hEGFR-D3 [1, 2]	GCTGTATCGCTGCAAGAAGC
	hEGFR-D1 [2, 3]	CGAGTACCTCATCCCACAGC
	hEGFR-ITD2 [2]	CTGGGTGTAAGAGGCTCCAC
***BRAF*-ID**	BRAF-c1 [1, 3]	GTGGGGAGCATAATCCACCA
	BRAF-c2 [1]	GGGCCTCTATATGTTCCTGTGT
	BRAF-c4 [1]	GTTGATCCTCCATCACCACGA
	BRAF-c5 [1, 2]	TCAGTTCCGTTCCCCAGAGA
	BRAF-c1b [2, 3]	GGGAGCATAATCCACCATCAATATATC
	BRAF-c4b [2]	ACCACGAAATCCTTGGTCTCTAAT
***KIAA1549-BRAF* fusion**	KIAA1549-e15 [1]	CTCAGCCTACATCGGATGCC
	KIAA1549-e16 [1]	AGACGGCCAACAATCCCTG
	BRAF-e9 [1]	GGGGGTAGCAGACAAACCTG
	BRAF-e11 [1]	CTGCCCATCAGGAATCTCCC
	KIAA1549-contr [1]	AGTAGATCCCTCTGGCAGCA
LMNA-NTRK1	LMNA-e2 [1]	TCTCAGTGAGAAGCGCACGC
	NTRK1-e13 [1]	GATGTCCCGGCGCTTGATGT
	LMNA-e7 [1]	AGGAGGTGGATGAGGAGGGC
ETV6-NTRK3	ETV6-3 [1, 3]	TCATGGTCTCTGTCTCCCCG
	NTRK3-2 [1]	TCGCTTCAGCACGATGTCTC
	ETV6-6 [1, 2]	CGGCTGTCAGAAAGCAACTG
	ETV6-5 [2]	GCCTGAAGAGCACGCCAT
	NTRK3-3 [2]	TCTCTCCTCTTAATGTGCTGCA

[1] used for RT-PCR

[2] used for subsequent nested RT-PCR of FFPE derived RNA

[3] used for sequencing
