# Supplementary material for: Recurrent intragenic rearrangements of EGFR and BRAF in soft tissue tumors of infants
Source: Nat Commun. 2018 Jun 18;9:2378. doi: 10.1038/s41467-018-04650-6 (PMC6006309; doi:10.1038/s41467-018-04650-6)
Supplement: Supplementary file 3 — Description of Additional Supplementary Files [file 41467_2018_4650_MOESM3_ESM.pdf]

Description of Supplementary Data Files

File Name: Supplementary Data 1
Description: List of all discovery and validation cohort tumor samples summarizing clinical information, sequencing assays performed and oncogenic rearrangements identified.

File Name: Supplementary Data 2
Description: Somatic point mutations identified by whole genome sequencing in the discovery cohort comprising 10 primary classical CMN tumors.

File Name: Supplementary Data 3
Description: Details of each oncogenic gene fusion detected in 17 CMN interrogated by whole genome sequencing (discovery and extension cohort).
